# Do Not Lose Your Head over the Unequal Regeneration Capacity in Prolecithophoran Flatworms

**DOI:** 10.3390/biology11111588

**Published:** 2022-10-28

**Authors:** Alexandra L. Grosbusch, Philip Bertemes, Bob Kauffmann, Clemens Gotsis, Bernhard Egger

**Affiliations:** Institute of Zoology, University of Innsbruck, 6020 Innsbruck, Austria

**Keywords:** Turbellaria, Prolecithophora, regeneration, evolution, non-model organism

## Abstract

**Simple Summary:**

Some flatworms can regenerate all body parts, including the head, while others cannot. We have studied the regeneration capacity of several prolecithophoran flatworm species to gain insights into the evolution of regeneration in flatworms, particularly the adiaphanidan clade, which includes the well-known planarians. We found that regeneration capacity greatly varies between different species and also between different families of Prolecithophora. No prolecithophoran was found to be able to regenerate the complete head. This suggests that planarians have likely evolved their head regeneration capacity de novo.

**Abstract:**

One of the central questions in studying the evolution of regeneration in flatworms remains whether the ancestral flatworm was able to regenerate all body parts, including the head. If so, this ability was subsequently lost in most existent flatworms. The alternative hypothesis is that head regeneration has evolved within flatworms, possibly several times independently. In the well-studied flatworm taxon Tricladida (planarians), most species are able to regenerate a head. Little is known about the regeneration capacity of the closest relatives of Tricladida: Fecampiida and Prolecithophora. Here, we analysed the regeneration capacity of three prolecithophoran families: Pseudostomidae, Plagiostomidae, and Protomonotresidae. The regeneration capacity of prolecithophorans varies considerably between families, which is likely related to the remaining body size of the regenerates. While all studied prolecithophoran species were able to regenerate a tail-shaped posterior end, only some Pseudostomidae could regenerate a part of the pharynx and pharynx pouch. Some Plagiostomidae could regenerate a head including the brain and eyes, provided the roots of the brain were present. The broad spectrum of regeneration capacity in Prolecithophora suggests that head regeneration capacity is not an apomorphy of Adiaphanida.

## 1. Introduction

Research on the regeneration capacity in flatworms begun with the discovery that some representatives of the Tricladida (‘planarians’) are able to fully regenerate every missing body part after amputation [1]. Ever since, triclads are known as the champions of regeneration [2,3,4,5]. Many studies have been undertaken to fully understand the mechanisms of the regeneration process in Tricladida, and much progress has been made in deciphering the signalling pathways that control the main body axis [6,7,8,9,10,11,12,13,14]. Among other things, manipulation of the canonical Wnt signalling pathway showed that the latter is involved in head regeneration and in positioning of the pharynx during regeneration [8,9,10,11]. However, many questions are still unanswered. Next to the studies performed in Tricladida, the regeneration capacity and stem cell dynamics during regeneration have already been determined in a number of other free-living flatworm taxa [15,16,17,18,19]. In general, three main types of regeneration capacity can be distinguished in free-living flatworms. There are two extremes, some species can regenerate all organs, including the head, such as most Tricladida and some Catenulida and Macrostomorpha, and other species cannot regenerate at all, such as many Rhabdocoela. Most species lie in between these extremes and are able to regenerate some organs from the posterior body part such as gonads and gut, but they cannot regenerate organs from the anterior body part such as brain and eyes [20]. Unfortunately, for many flatworm groups, only limited data on regeneration capacity are available.

**Figure 1:**
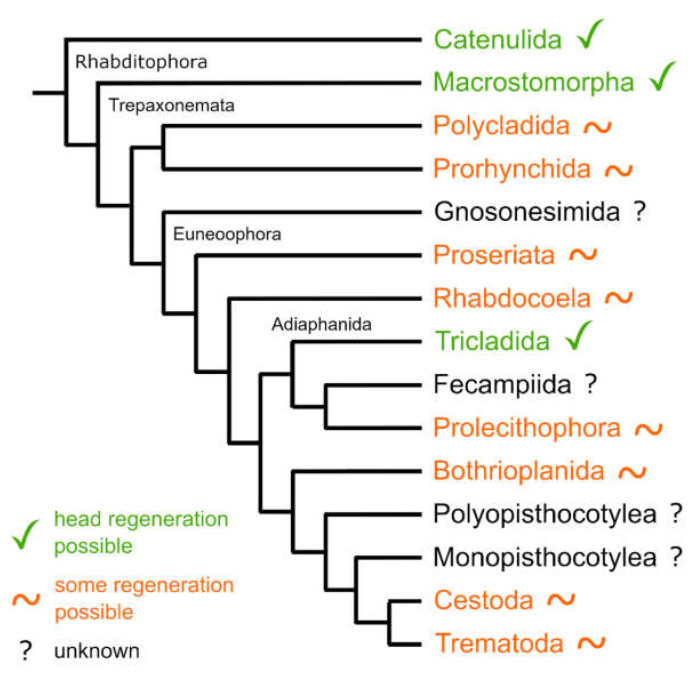
Inter-relationships of higher-level taxa in Platyhelminthes, drawn after [24,26,27]. Green colour and check marks indicate that at least some representatives of the taxon are known to completely regenerate a head. Orange colour and tilde indicate that at least some representative of the taxon are known to regenerate some body parts, but never the complete head. Black colour and a question mark indicate that no regeneration studies have been undertaken for the respective taxa.

Tricladida, together with Prolecithophora and Fecampiida, form the clade Adiaphanida [21] (Figure 1). While the regeneration capacity in Tricladida is well studied, the regeneration abilities of Prolecithophora are poorly understood [20]. There are only two published studies about the regeneration capacity of prolecithophorans, which are contradictory in their findings and vague in their descriptions. On one hand, according to Keller [22], the freshwater species *Plagiostomum lemani* has only poor regeneration powers. On the other hand, Monti [23] reports excellent regeneration capacity for its congener, *Plagiostomum girardi*. As a possible sister group to triclads [24,25], prolecithophorans are a most interesting target to study whether a key regeneration mechanism present in planarians is also present in prolecithophorans.

The taxon Prolecithophora is only poorly studied and no model organisms are available. They are tiny (within the millimetre range), mostly drop-shaped free-living flatworms, which are very common on algae and soft sediments of marine habitats [28,29,30]. As a member of the Adiaphanida (from the Greek word adiafanis which means opaque), they all have a non-transparent epidermis [19]. Most representatives have a ciliary groove and two pairs of eyes in the anterior body part at the level of the brain. The morphology and location of the pharynx and genital organs vary greatly, but they present an important feature for species determination. Three different types of pharynges are described within prolecithophorans—pharynx simplex, pharynx plicatus and pharynx variabilis. The pharynx can be situated in the anterior or posterior body half and orientated anteriorly, ventrally or posteriorly. As prolecithophorans are hermaphrodites, they have male and female genital organs, which often have a common opening with the pharynx (orogenital opening) at the ventral body side. Seminal vesicles and ovaries may be paired or unpaired. Furthermore, germaries and vitellaries are either separated or build the germovitellaries [31,32,33,34]. Currently, the large neoophoran flatworm taxon comprises five families: Multipeniatidae, Plagiostomidae, Protomonotresidae, Pseudostomidae and Scleraulophoridae. The families show a great difference between number of species. The two largest families are Plagiostomidae (nine genera with over 100 species) and Pseudostomidae (12 genera with over 50 species) followed by Protomonotresidae (seven genera with 14 species), Multipeniatidae (one genus with four species) and Scleraulophoridae (one genus with only two species) [35,36].

**Figure 2:**
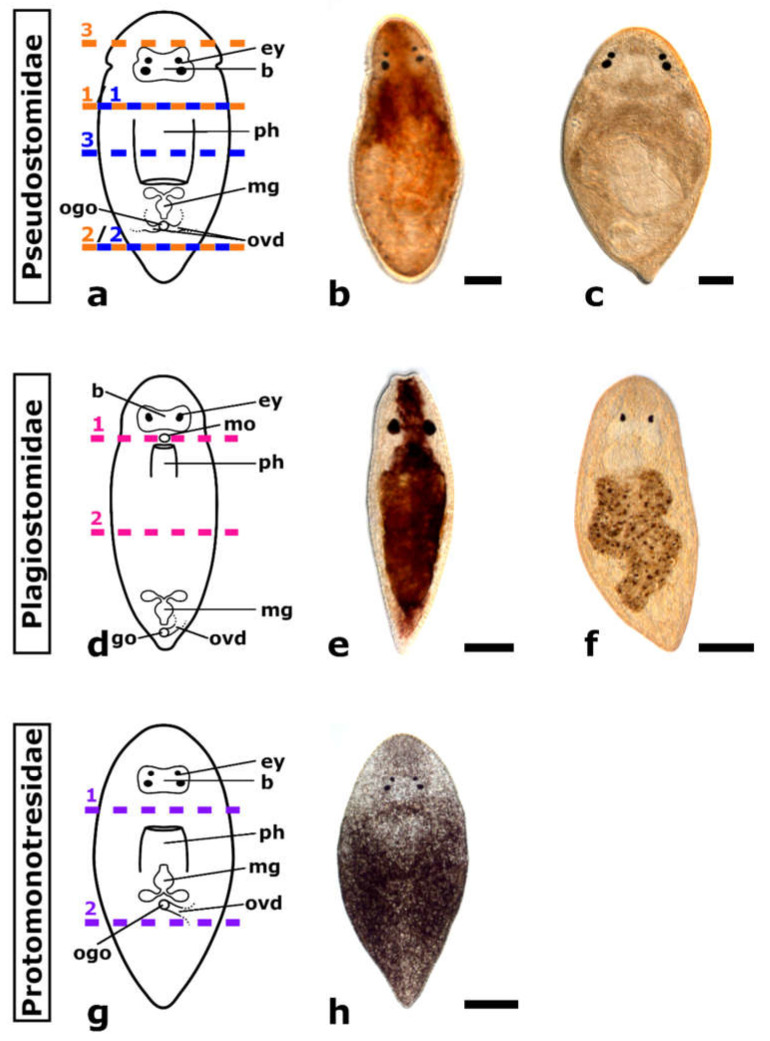
The five prolecithophoran species studied in detail belonging to three different families (Pseudostomidae, Plagiostomidae, Protomonotresidae). Schematic drawings of a Pseudostomidae (**a**), a Plagisostomidae (**d**) and a Protomonostresidae (**g**), showing the different amputation levels. The amputation levels used for each species are indicated in a different colour: *Monoophorum striatum* in orange, *Cylindrostoma monotrochum* in blue, *Vorticeros auriculatum* and *Plagiostomum girardi* in pink, and Protomonostresidae sp. in purple. Differential contrast images of living squeeze-prepped animals of *Monoophorum striatum* (**b**), *Cylindrostoma monotrochum* (**c**), *Vorticeros auriculatum* (**e**), *Plagiostomum girardi* (**f**), and Protomonostresidae sp. (**h**). Abbreviations: b, brain; ey, eye; go, genital opening; mg, male genital organ; mo, mouth opening; ogo, orogenital opening; ovd oviduct; ph, pharynx. Scale bars: 100 µm in (**b**,**c**,**f**); 200 µm in (**e**,**h**).

Even though Plagiostomidae is the family with most described species, it also presents the least morphological differences. Plagiostomidae have an elongated body shape and rarely have a ciliated groove in the area of the brain. They have mostly one pair of eyes and no distinct brain capsule. The mouth and genital opening are separated. The anteriorly directed pharynx lies in the anterior body part either in front or right behind the brain and opens through the mouth on the ventral side of the anterior body part or at the tip of the anterior body end. The male copulatory organ as well as the common genital opening lie in the posterior body part [32,37] (Figure 2d).

Protomonotresidae have a compact body shape without a ciliated groove and, except for a single genus (Prorogonophora), they are all described to lack eyes. The anteriorly or ventrally directed pharynx and male copulatory organ lie in the anterior body part close to the middle and mostly open through an orogenital opening on the ventral side of the anterior body part. Only one genus (Baicalarctia) has a separated mouth and genital opening on the ventral side of the anterior body part [38,39] (Figure 2g).

Pseudostomidae show a quite diverse morphology. Mostly, their body shape is similar to that of Protomonotresidae, but some are more oblong and thus more similar to Plagiostomidae. They have one, rarely two ciliated grooves close to the front end of the body and zero to three pairs of eyes. However, they all have a distinct brain capsule. Except for the genus *Ulianinia*, which has the mouth and genital opening separated, Pseudostomidae have an orogenital opening. Generally, the pharynx and the male copulatory organ lie in the posterior body half or in mid body and point either to the front or the hint end of the body. Few exceptions are known, in the genera Gonostomula and Reisingeria, the pharynx and the male copulatory organ lie in the anterior body part near the front end where the orogenital opening is located [32,37] (Figure 2a).

With this study, we first provide profound key points on otherwise scarce knowledge on the regeneration capacity in the flatworm taxon Prolecithophora. The regeneration capacity of 13 different prolecithophoran species belonging to three different families (Pseudostomidae (Figure 2a), Plagiostomidae (Figure 2d) and Protomonotresidae (Figure 2g)) were studied. The main focus of the present work lies on five different species (*Monoophorum striatum* (Figure 2b), *Cylindrostoma monotrochum* (Figure 2c), *Vorticeros auriculatum* (Figure 2e), *Plagiostomum girardi* (Figure 2f) and *Protomonotresidae* sp. (Figure 2h)) for which the regeneration process is described in detail. With these new data, we want to explore whether the regeneration capacity in Tricladida is an autapomorphy or a plesiomorphy of Tricladida retained from an adiaphanidan ancestor.

## 2. Materials and Methods

### 2.1. Specimen Collection, Determination and Maintenance

Prolecithophoran specimens were collected during field trips to the small town Punat on the Croatian island Krk (45°01′23″ N 14°37′41″ E) in March and October 2016, March and October 2017, May 2018, October 2019, as well as May, June and October 2021. They were extracted from brown algae in the laboratory with 1:1 7.14% MgCl_2_ × 6H_2_O and seawater solution and were collected with Pasteur pipettes under a stereo microscope. Species determination of all 13 studied species (four Pseudostomidae: *Cylindrostoma monotrochum, Cylindrostoma* sp., *Monoophorum striatum*, *Monoophorum* sp.; eight Plagiostomidae: *Acmostomum dioicum*, *Plagiostomum chromogastrum*, *Plagiostomum* sp., *Plagiostomum girardi*, *Plagiostomum koreni*, *Plagiostomum maculatum*, *Plagiostomum morgani*, *Vorticeros auriculatum* and one Protomonotresidae: Protomonotresidae sp.) was performed using histological sections. Morphological characteristics of Protomonotresidae sp. and the fact that they have two pairs of eyes indicate that it is most likely a Prorogonophora species. In the field, they were kept in Petri dishes with seawater (3.5–3.7%) at room temperature, ranging from 19 to 23 °C. In laboratory conditions, specimens were kept alive in Petri dishes containing 3.5% artificial seawater (ASW) in a climate chamber, at 18 °C with 60% humidity and a 14:10 h day–night cycle without feeding for up to one month, until they were used for experiments.

### 2.2. Amputation and In Vivo Observation

Mature specimens were amputated at different body levels, to determine the regeneration capacity of the different species. The first amputation level was chosen to examine if the anterior part (=posterior regenerate) is able to regenerate the tail with the pharynx and if the posterior part (=anterior regenerate) is able to regenerate a head with brain and eyes (Figure 2a,d,g amputation level 1). Another amputation level was selected to test if the posterior regenerate, not deprived of brain and pharynx, can regenerate a tail and if the anterior regenerate can regenerate the head with the brain and pharynx (Figure 2a,d,g amputation level 2). An additional amputation level was studied in some Pseudostomidae species. In *M. striatum*, an amputation just in front of the brain was performed to check if they are able to restore the shape of the head (Figure 2a amputation level 3 in orange). In *C. monotrochum* and *Cylindrostoma* sp., an amputation level through the middle of the pharynx was chosen to analyse regeneration of the pharynx (Figure 2a amputation level 3 in blue).

Experiments were performed in two different locations. One part was carried out in the marine biological station of the marine conservation organisation MareMundi in Punat (Krk, Croatia) near the sampling location. The other part was carried out in the laboratory at the University of Innsbruck after the collected animals had been transported to Austria.

All amputations were performed on a glass slide in a small droplet of either 3.5% ASW (or filtered seawater in the field) or 1:1 7.14% MgCl_2_ × 6H_2_O in 3.5% ASW (or filtered seawater in the field) solution with a razor blade (Gillette, WY, USA) under a stereo microscope. Amputated animals were transferred to Petri dishes containing either filtered seawater or 3.5% ASW. Regenerates were maintained in a climate chamber at 18 °C with 60% humidity and a 14:10 h day–night cycle without feeding (or at room temperature and the natural day–night cycle in the field). The amputated animals were observed every second day during three weeks under a stereo microscope to check for changes of their outer shape and their behaviour. Live squeeze preparations were performed at least every two days to take pictures of the different regeneration stages. Live animals were transferred back to the Petri dishes after squeezing, so further development of the same individual could be observed.

### 2.3. Immunocytochemistry and Fluorescent Staining

To analyse the extent to which Plagiostomidae are able to regenerate a brain, the serotonergic nervous system in posterior and anterior regenerates of *V. auriculatum* was stained with an antibody against 5-hydroxytryptamine (5-HT). Herefore, we amputated 16 individuals between brain and pharynx and treated them separately. Posterior regenerates were fixed 24 h post amputation while anterior regenerates were left to regenerate for 12 days. Posterior and anterior regenerates were anaesthetised for 20–30 min with 7.14% MgCl_2_ × 6H_2_O under visual control. Subsequently, they were fixed for 1 h at room temperature (RT) in 4% formaldehyde in 0.1 M PBS (phosphate buffered saline). After washing for 1 h with PBS, specimens were dehydrated in a gradually ascending ethanol series (25%, 50%, 75%, 90%, and 100%) and stored at −20 °C. Right before the staining, specimens were rehydrated in gradually descending ethanol concentrations (100%, 90%, 75%, 50%, 25%), bleached in 6% H_2_O_2_ solution in PBS-T_x_ (1× phosphate buffered saline with 0.1% Triton X-100, Sigma-Aldrich, St. Louis, MO, USA) overnight under bright light, and subsequently rinsed several times with PBS-T_x_ for 1 h. Then, specimens were incubated for 2 h at RT in BSA-T_x_ (PBS-T_x_ with 1% bovine serum albumin, Carl Roth, Karlsruhe, Germany) for blocking and then overnight at 4 °C in the primary antibody rabbit-anti-5-HT (Sigma-Aldrich) diluted 1:2000 in BSA-T_x_. Next, specimens were rinsed several times with PBS-T_x_ for 2 to 3 h, blocked with BSA-T_x_ for 2 h and incubated in a secondary fluorescein isothiocyanate-conjugated (FITC) goat anti-rabbit antibody, diluted 1:250, for 2 h in the dark, each at RT. Before mounting specimens in either Vectashield (Vector Laboratories, Newark, CA, USA) or Aqua-Poly/Mount (Polysciences, Warrington, PA, USA), they were washed with PBS-T_x_ at least 80 times during 4 to 5 days (at RT) and 4 to 5 nights (at 4 °C) in darkness.

To study pharynx regeneration in posterior regenerates of *V. auriculatum* and *C. monotrochum*, muscle actin filaments were visualised with phalloidin. The experimental set up was similar to that explained in the paragraph above, with the only difference that here the anterior regenerate was fixed 24 h post amputation and the posterior regenerate was left to regenerate. Fixation of posterior and anterior regenerates was performed as mentioned in the paragraph above. Then, they were washed several times with PBS for 1 h and stored at 4 °C for a few weeks. After a short washing step with PBS-T_x_, specimens were incubated first in BSA-T_x_ for 2 h and then in Atto-565-conjugated phalloidin (Sigma-Aldrich), diluted to 1:250 in BSA-T_x_ for 2 h. Finally, they were rinsed and mounted as described above.

### 2.4. Histological Sections and Stainings

Specimens were relaxed, fixed and dehydrated as described above. They were stored for several weeks in 100% ethanol. Next, they were transferred to acetone and subsequently infiltrated for one night with a 1:1 acetone/EPON mixture. Then, they were transferred to 100% EPON for another night before they were transferred to an embedding form filled with 100% EPON. After polymerisation of the resin, sagittal and horizontal semi-thin sections at 3 µm thickness were made. A standard protocol for toluidine blue [40] was used to stain the sections.

### 2.5. Microscopy and Visualisation

Live observations, amputations and handling animals were performed under either a Nikon SMZ645 or a Nikon SMZ-2B stereo microscope. Pictures of live squeeze preparations were taken using either a Leica DM 5000 B microscope equipped with a Leica DFC 490 camera or on a Leitz Diaplan light microscope equipped with an The Imaging Source DFK 33UX264 camera. A Leica TCS SP5 II confocal microscope was used to make confocal stacks of fluorescently stained animals. Figure composition and schematic drawings were performed with the open-source software GIMP up to v. 2.10.30 [41] and Inkscape up to v. 1.0.1 [42]. Confocal stacks were processed with the open-source program Fiji up to v. 1.53g [43]. Depth colour-coded images were created with Fiji by using the included look up tables “Ice”, “mpl plasma” and “ICA 3”.

### 2.6. Molecular Phylogeny

DNA or RNA from animals were extracted, sequenced and assembled for obtaining partial marker molecules 18S (nuclear small ribosomal subunit) and 28S (nuclear large ribosomal subunit) as described in [44] and [26]. Additional prolecithophoran sequences were downloaded from Genbank. Gene sequences were separately aligned for 18S and 28S sequences using MUSCLE v3.8.31 [45]. The resulting alignments were manually trimmed for a length of 1747 nt (18S) and 1308 nt (28S) and concatenated for a total length of 3055 nt. Phylogenetic reconstruction was performed with IQ-TREE v2.1.3 [46] using gene partitions, searching for the best models (GTR + G + I for both partitions) and calculating 1000 bootstrap pseudo-replicates. The resulting consensus tree was edited with FigTree v1.4.2 [47] and Inkscape up to v. 1.0.1 [42].

## 3. Results

### 3.1. Regeneration Capacity in Pseudostomidae

#### 3.1.1. Amputation Anterior to the Brain

**Figure 3:**
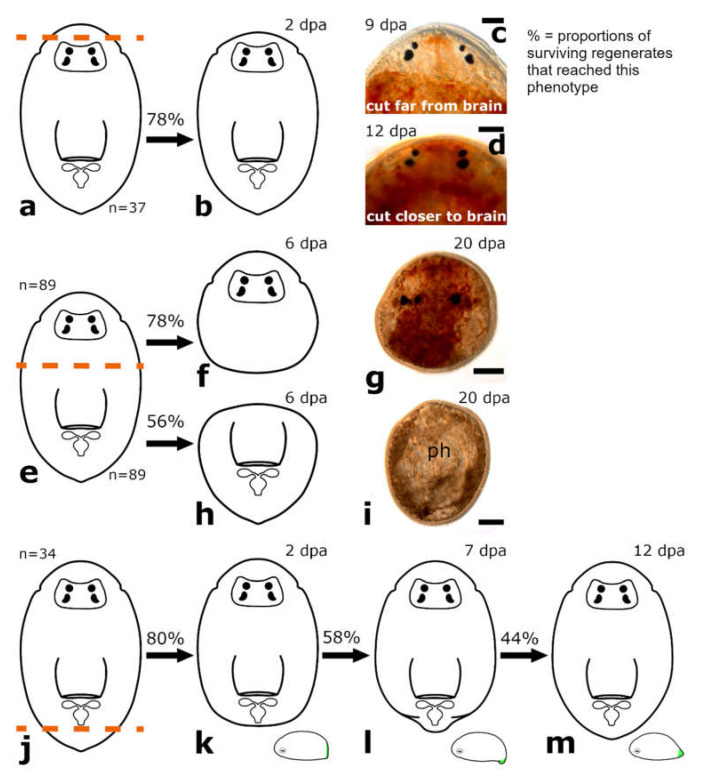
Regeneration in *Monoophorum striatum*. Amputation anterior to the brain (**a**); anterior regenerates fully healed the wound (**b**). The amputated tissue was not restored at any time (**c**,**d**). Amputation between brain and pharynx (**e**); complete wound healing in posterior regenerates (**f**,**g**) and anterior regenerates (**h**,**i**); no further regeneration was observed. Amputation posterior to the pharynx (**j**); posterior regenerates fully healed the wound (**k**). The regenerated tissue was bent towards the ventral side (**l**). The newly build tail tip was not retracted anymore (**m**). Abbreviations: mg, male genital organ; ph, pharynx. Scale bars: 50 µm in (**c**,**d**); 100 µm in (**g**,**i**).

The amputation level anterior to the brain was performed only on specimens of *Monoophorum striatum* (*n* = 37) (Figure 3a).

##### Posterior Regeneration

Posterior regenerates were not able to regenerate at all. Most of them disintegrated immediately after the amputation, with the longest-lasting regenerate making it no further than 6 days post amputation (dpa).

##### Anterior Regeneration

In anterior regenerates, wound healing was completed after 2 dpa in 78% of the anterior regenerates (Figure 3b). Individuals amputated at a greater distance from the brain showed a more roundish shape (Figure 3c) at the amputation site than those amputated closer to the brain (Figure 3d). Observation of the amputees went on until 19 dpa with only 10% of the anterior regenerates still alive (Figure S1b). However, the missing tissue was not rebuilt and no blastema-like structure was observed at any time (Figure 3c,d).

#### 3.1.2. Amputation between Brain and Pharynx

**Figure 4:**
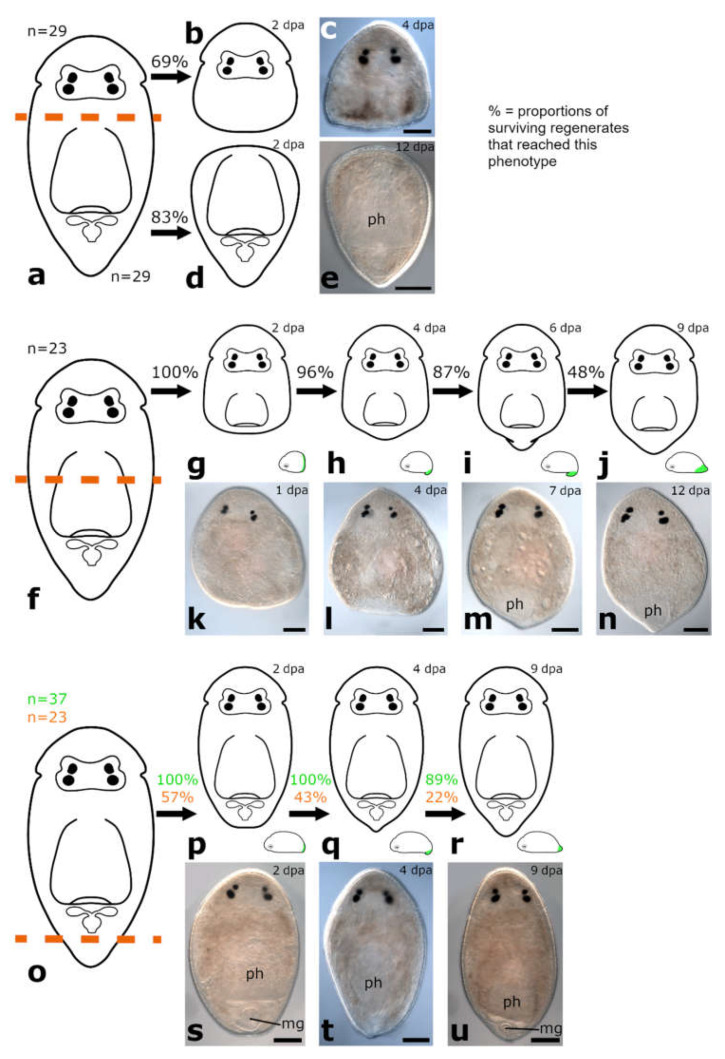
Regeneration in *Cylindrostoma monotrochum*. Amputation between brain and pharynx (**a**): complete wound healing in posterior regenerates (**b**,**c**) and anterior regenerates (**d**,**e**); no missing body parts were regenerated. Amputation through the middle of the pharynx (**f**): posterior regenerates fully healed the wound (**g**,**k**). Few days later, an unpigmented, blastema-like structure, which was bend towards the ventral side (**h**,**l**), and continuously grew and tapered (**i**,**m**) was observed. The newly built tail tip was no longer bent to the ventral side (**j**,**n**). Amputation posterior to the pharynx (**o**): complete wound healing in posterior regenerates in the field (green) and under laboratory conditions (orange) (**p**,**s**). A blastema-like structure, which was bend towards the ventral side was build (**q**,**t**), and led to a complete regeneration of the missing tail tip (**r**,**u**). Same individual shown in (**k**–**n**). Abbreviations: mg, male genital organ; ph, pharynx. Scale bar is 100 µm.

The amputation level between brain and pharynx was performed on specimens of *Monoophorum striatum* (*n* = 89) and *Cylindrostoma monotrochum* (*n* = 29) (Figure 3e and Figure 4a).

##### Posterior and Anterior Regeneration in *Monoophorum striatum*

Posterior and anterior regenerates of *M. striatum* could not regenerate a tail or a head, respectively (Figure 3f–i). Only after 6 dpa, in 78% of the posterior regenerates and 56% of the anterior regenerates, the wound was healed. No blastema-like structure was observed in the following two weeks of observation (Figure 3h,i). Among the anterior regenerates, ca. 20% of specimens had the pharynx stretched out and pointed towards the anterior and they were not able to retract it anymore. Therefore, the wound could not fully heal. Nearly 30% of the amputees of *M. striatum* survived more than three weeks without any sign of regenerating any organ, yet they were still able to swim around in the Petri dish (Figure S1a,b).

##### Posterior and Anterior Regeneration in *Cylindrostoma monotrochum*

Posterior and anterior regenerates of *C. monotrochum* were also not able to regenerate the missing body parts (Figure 4b–e). However, in 69% of posterior regenerates and 83% of anterior regenerates, wound healing was already completed after 2 dpa. Nevertheless, the shape of the wound site did not change during the observation period and neither brain nor pharynx regeneration was observed. The survival rate of the animals amputated and observed in the remote laboratory (Innsbruck, Austria) was lower than those amputated and observed directly in the field (in the premises of the marine station in Punat, Croatia) (Figure S1c,d). While all posterior regenerates of both batches were dead after 12 days, for anterior regenerates this was only the case for the batch studied in the remote laboratory. From the batch studied in the field, 90% of anterior regenerates were still alive after 12 days, although without showing any sign of regeneration.

##### Posterior and anterior Regeneration in Other Pseudostomids

Two additional pseudostomid species (*Monoophorum* sp. (*n* = 10) and *Cylindrostoma* sp. (*n* = 10)) were likewise amputated between brain and pharynx. The observed regeneration process of posterior and anterior regenerates of both species correlated with that of their respective relatives, i.e., they did neither regenerate a head, nor a tail (data not shown).

#### 3.1.3. Amputation through the Pharynx

The amputation level through the pharynx was analysed on two different *Cylindrostoma* species, *Cylindrostoma* sp. (*n* = 180) and *Cylindrostoma monotrochum* (*n* = 23) (Figure 4f).

##### Posterior Regeneration

**Figure 5:**
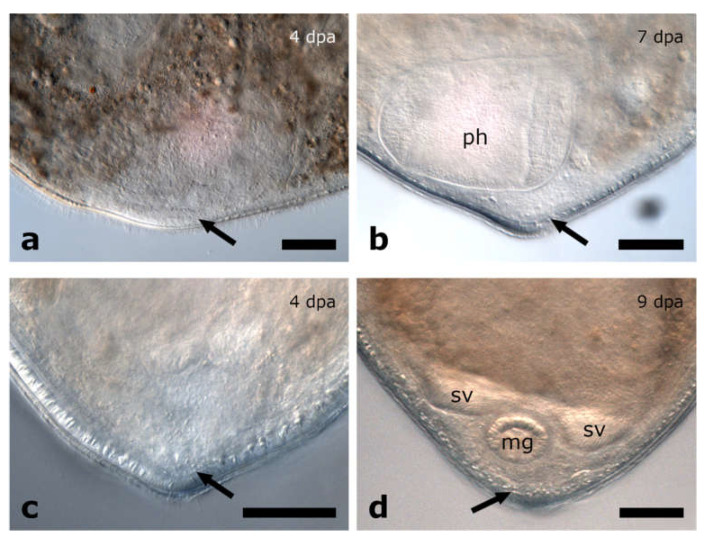
Blastema-like tissue in posterior regenerates of *Cylindrostoma monotrochum*. After being amputated through the middle of the pharynx (**a**,**b**) or posterior to the pharynx (**c**,**d**), an unpigmented, blastema-like structure was formed at the wound site (**a**,**c**). The structure continued growing and finally build a new pointy tail tip (**b**,**d**). Arrows are pointing at the blastemal-like tissue. Abbreviations: mg, male genital organ; ph, pharynx; sv, seminal vesicle. Scale bar is 50 µm.

**Figure 6:**
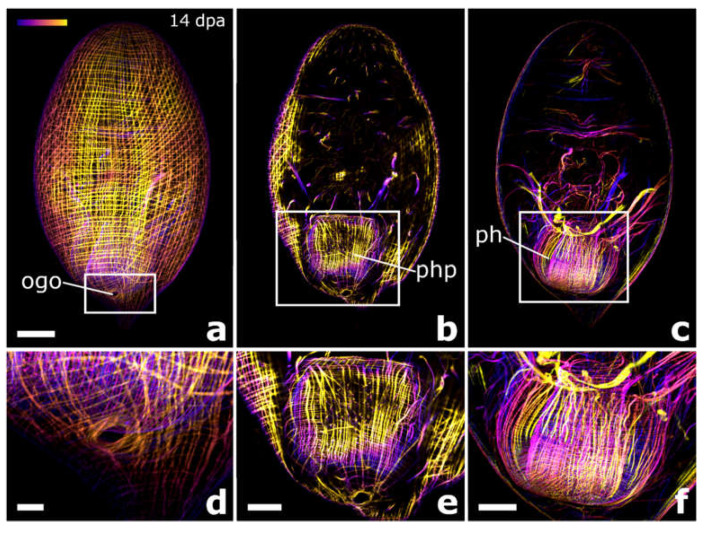
Musculature in posterior regenerates of *Cylindrostoma monotrochum*. F-actin stainings of a posterior regenerate 14 days after amputation through the pharynx. The regenerated orogenital opening at the posterior ventral part of the animal (**a** and in detail in **d**), the regenerated pharynx pouch (**b** and in detail in **e**), and the regenerated pharynx (**c** and in detail in **f**). The depth colour-coded projections show the same individual as in Figure 4k–n. Blue is more dorsal and yellow is more ventral in all pictures. Abbreviations: ogo, orogenital opening; ph, pharynx; php, pharyngeal pouch. Scale bars: 50 µm in (**a**) (same for **b** and **c**); 10 µm in (**d**) and 25 µm in (**e**,**f**).

*Cylindrostoma* sp. was amputated in mid-body, leaving some posterior regenerates with a short piece of the pharynx root, and some without. Finally, 27 of the posterior regenerates were observed to have regenerated a tail and part of the pharynx after 16 dpa (data not shown). Specimens of its congener *Cylindrostoma monotrochum* were amputated through the pharynx to further analyse pharynx regeneration in Pseudostomidae. All (100%) of the posterior regenerates were able to heal the wound completely within two days (Figure 4g,k). Four days after amputation, an early blastema-like structure, which was bent towards the ventral side and thus only visible in actively swimming animals, was formed in 96% of the regenerates (Figure 4h,l and Figure 5a). After 6 dpa, the blastema-like structure had grown and tapered in 87% of the regenerates, but was still retracted to the ventral side (Figure 4i,m and Figure 5b). After approximately 9 dpa, 48% of the posterior regenerates were observed to swim around in the Petri dish fairly lively, and the regenerated yet still unpigmented tail tip was no longer retracted (Figure 3j,n). However, the shape of the newly built tail tip was not as pointed as in a wild-type animal. As the animals are quite opaque, regeneration of the pharynx could not be studied during live observations in the stereo microscope. Therefore, we performed phalloidin staining on 13 individuals to visualise the potential regeneration of the pharynx based on the musculature. The staining revealed that posterior regenerates were able to restore the shape of the pharyngeal pouch with a length of ca. 98 +/− 7.8 µm (*n* = 7) (Figure 6b,e) and the pharynx with a diameter of ca. 99 +/− 7.5 µm (*n* = 5) (Figure 6c,f). Additionally, the combined mouth and genital opening was fully regenerated (Figure 6a,d).

##### Anterior Regeneration

In anterior regenerates, wound healing was observed after 2 dpa. No changes in shape were observed during the following ten days of live observations (data not shown).

#### 3.1.4. Amputation Posterior to the Pharynx

The amputation level posterior to the pharynx was performed in specimens of *Monoophorum striatum* (*n* = 34) and *Cylindrostoma monotrochum* (*n* = 37 in the field and *n* = 23 in the remote laboratory) (Figure 3j and Figure 4o).

##### Posterior Regeneration

In *M. striatum*, approximately 80% of posterior regenerates closed the wound at 2 dpa (Figure 3k). It took them approximately one week to build a blastema-like structure, that was bend towards the ventral side (Figure 3l). This structure was observed in 58% of posterior regenerates. As the regenerates were still fragile at this time, it was not possible to take good pictures of the regenerated tissue without harming them. Additionally, two of the posterior regenerates were not able to retract the pharynx which was stretched out posteriorly after the amputation, thus preventing them from forming a new tail tip. The newly built tail tip was not retracted anymore at 12 dpa in 44% of posterior regenerates (Figure 3m). Still, the pointy shape was more blunt compared to wild-type animals. While all amputation experiments of *M. striatum* were performed in the laboratory in Innsbruck, those of *C. monotrochum* were partly performed in the remote laboratory in Innsbruck and partly in the field. In *C. monotrochum*, a great variation in the number of regenerates reaching different stages was noticed between animals treated in the field and in the remote laboratory. While 100% of the regenerates cut and observed in the field already showed a fully healed wound after 1 dpa, only 57% of the regenerates treated in the remote laboratory closed the wound at 2 dpa (Figure 4p,s). In 100% of posterior regenerates treated in the field and 43% of posterior regenerates studied in the remote laboratory, a blastema-like structure that was bent towards the ventral side could be observed at 4 dpa (Figure 4q,t and Figure 5c). Over the course of the following week of observation, the blastema-like structure gradually became more pointy, until at 9 dpa 89% of the regenerates treated in the field and 22% of those studied in the remote laboratory did not retract the newly built tail tip anymore (Figure 4r,u and Figure 5d). The number of surviving amputees of both species decreased over the course of observation time (Figure S1a–d). However, we observed a difference in the survival rates between experiments that were carried out in these two different locations. All the posterior regenerates resulting from amputations performed in the field were still alive after 4 dpa, whereas almost half of them were dead at that time point in the remote laboratory in Innsbruck. In the field, 72% of the posterior regenerates were still alive at 12 dpa, compared to 23% in the remote laboratory (Figure S1c).

##### Anterior Regeneration

In *M. striatum*, for most anterior regenerates, the remaining body size was very small in relation to the wounded tissue and were thus not able to contract the wound site and spilled out (data not shown). All anterior regenerates were dead by 6 dpa. In anterior regenerates of *C. monotrochum*, complete wound healing was observed after 2 dpa, when treated in the remote laboratory and in those studied in the field already after 1 dpa. No more events were observed in anterior regenerates (data not shown). It was again noticeable that the number of surviving amputees studied in the remote laboratory decreased faster than those studied in the field. All anterior regenerates observed in the remote laboratory were dead after 7 dpa, whereas 76% of the anterior regenerates studied in the field were still alive at this time point (Figure S1d). However, even in the field, animals quickly decreased in number from 7 dpa onwards, so that all but one amputee were dead at 12 dpa.

##### Posterior and Anterior Regeneration in Other Pseudostomids

Amputation posterior to the pharynx of *Monoophorum* sp. (*n* = 10) and *Cylindrostoma* sp. (*n* = 16) resulted in the same regeneration process of posterior and anterior regenerates as in *M. striatum* and *C. monotrochum* mentioned above, except that only three of the posterior regenerates of *Monoophorum* sp. were able to rebuild the tail tip (data not shown).

### 3.2. Regeneration Capacity in Plagiostomidae

#### 3.2.1. Amputation between Brain and Pharynx

**Figure 7:**
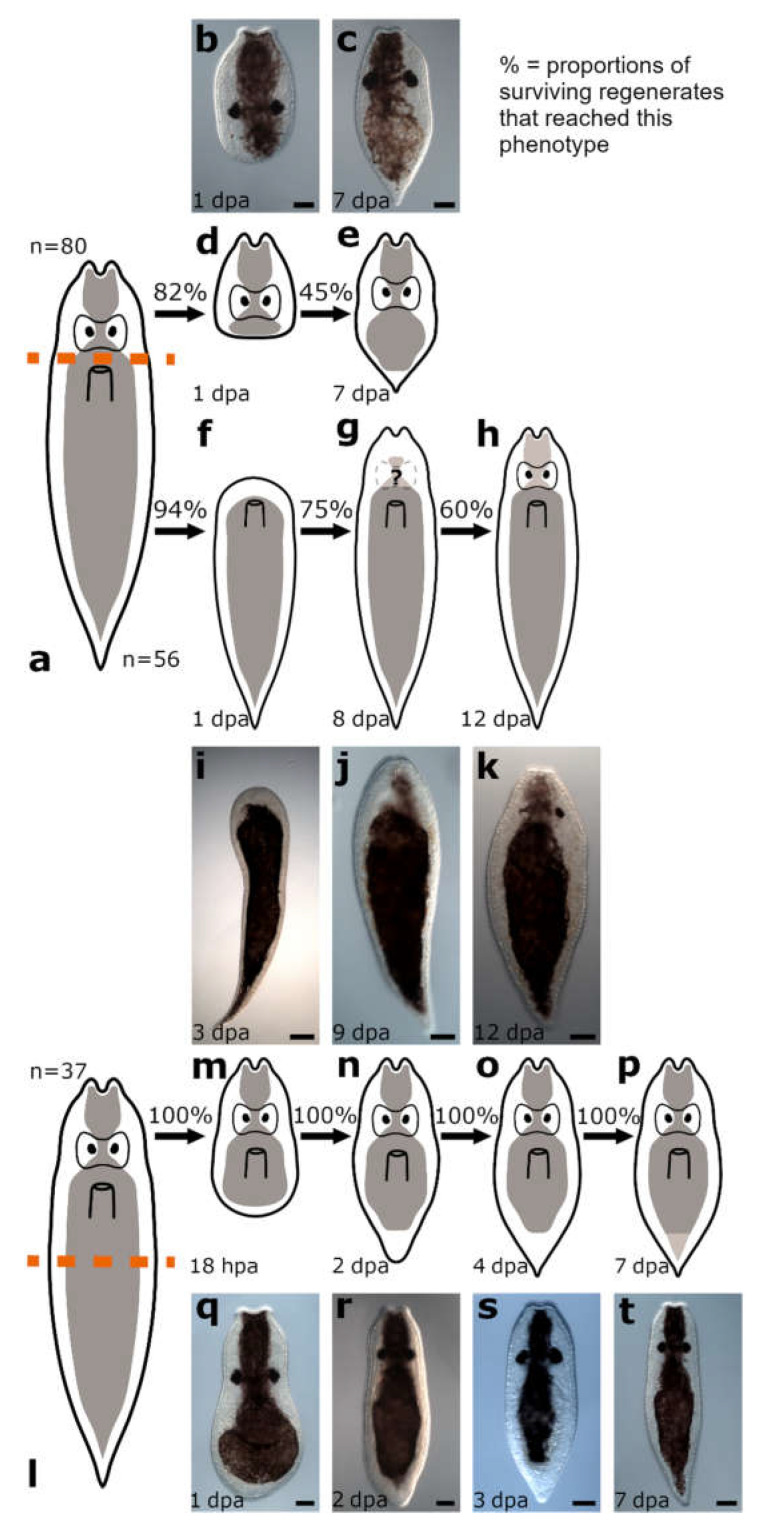
Regeneration in *Vorticeros auriculatum*. Amputation between brain and pharynx (**a**). Posterior regenerates fully healed the wound (**b**,**d**). A small tail was regenerated (**c**,**e**), but no pharynx primordium was clearly visible. Anterior regenerates fully healed the wound and started to form a blastema-like structure (**f**,**i**). The original head shape, including the peculiar anterior-facing tentacles, was restored (**g**,**j**). Eyes and pigmentation were regenerated in anterior regenerates (**h**,**k**). Amputation posterior to the pharynx (**l**). A small blastema-like structure became visible (**m**,**q**), a somewhat roundish tail started to form (**n**,**r**), developed over time into the original tail shape (**o**,**s**). Pigmentation in the new tail tip was restored (**p**,**t**). Scale bars: 50 µm in (**b**,**c**,**j**,**k**) and (**q**–**t**); 100 µm in (**i**).

**Figure 8:**
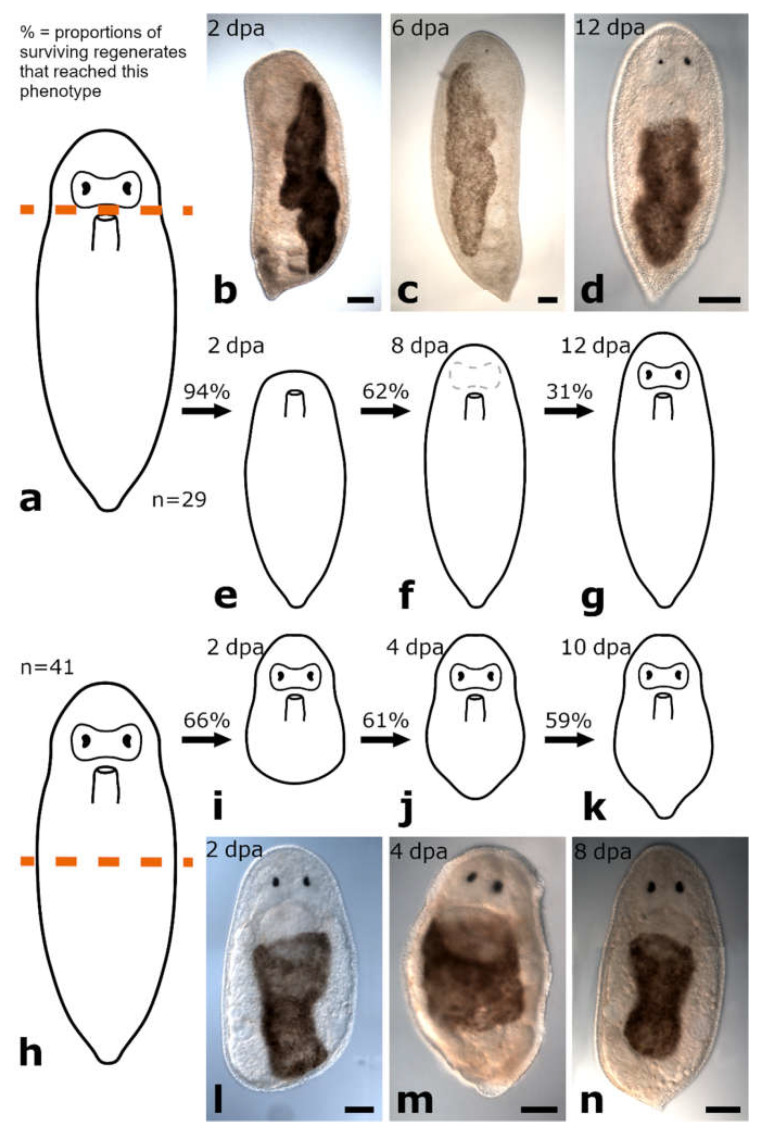
Regeneration in *Plagiostomum girardi*. Anterior regeneration after amputation between brain and pharynx (**a**–**g**). The wound was fully healed (**b**,**e**). The original head shape was restored (**c**,**f**). Eye regeneration (**d**,**g**). Posterior regeneration after amputation posterior to the pharynx (**h**–**n**). Complete wound healing (**i**,**l**). A small blastema-like structure was built at the incision site (**j**,**m**) and lead to the regeneration of a pointy tail was regenerated (**k**,**n**). Scale bars are 100 µm.

The amputation level between brain and pharynx was studied in *Vorticeros auriculatum* (*n* = 80 for posterior regenerates and *n* = 56 for anterior regenerates) and *Plagiostomum girardi* (*n* = 27 for posterior regenerates and *n* = 29 for anterior regenerates) (Figure 7a and Figure 8a).

##### Posterior Regeneration

Posterior regenerates of *V. auriculatum* completed wound healing after 1 dpa (Figure 7b,d), whereas it took posterior regenerates of *P. girardi* 2 dpa to achieve this (data not shown). This stage was reached by 82% of *V. auriculatum* and 92.5% of *P. girardi* specimens (Figure S2a,c). Posterior regenerates of *V. auriculatum* build a new tail tip at the wound site that doubled its size during the two weeks of observation until the tail returned to its original shape (Figure 7c,e). After 1 week, 45% of the amputees looked like miniatures of the intact animals and their behaviour was again like that of intact animals. It was also observed that the regenerates, like the wild-type animals, were able to attach themselves to the bottom of the Petri dish with the newly built tail tip. In contrast, no regeneration beyond wound healing was observed in posterior regenerates of *P. girardi*. However, the survival rate decreased steadily in both species until in *P. girardi* only 40% of the regenerates were still alive at 9 dpa and in *V. auriculatum* at 12 dpa (Figure S2a,c).

To test if the cutting level was accurate, we looked for the pharynx in both anterior and posterior regenerates by using phalloidin stainings (*n* = 10). We detected a pharynx only in either the anterior (*n* = 3) or the posterior regenerates (*n* = 7), but never in both, indicating that no pharynx was regenerated in any piece and that the cutting level was accurate in 70% of the attempts.

##### Anterior Regeneration

**Figure 9:**
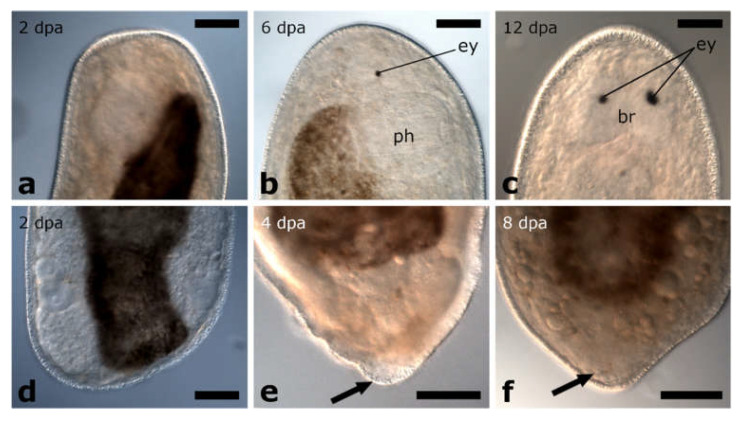
Blastema-like tissue in posterior and anterior regenerates of *Plagiostomum girardi***.** Amputation between brain and pharynx (**a**–**c**) lead to complete wound healing in anterior regenerates (**a**), followed by the restoration of the original head shape (**b**) and eye regeneration (**c**). After being amputated posterior to the pharynx (**d**,**e**), first, complete wound healing was observed in posterior regenerates (**d**). Then, a small, blastema-like structure was build (**e**) which grew to a pointy, new tail tip (**f**). Arrows are pointing at the blastema-like tissue. Abbreviations: br, brain; ey, eye; ph, pharynx. Scale bar is 100 µm.

**Figure 10:**
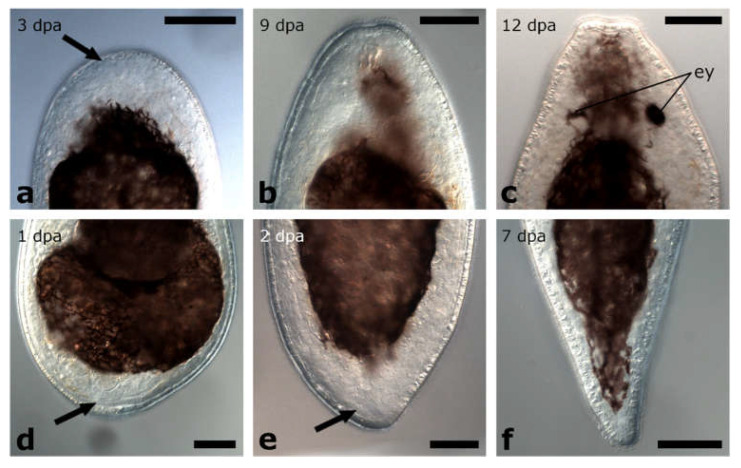
Blastema-like tissue in in posterior and anterior regenerates of *Vorticeros auriculatum*. Amputation between brain and pharynx (**a**–**c**) and amputation posterior to the pharynx (**d**–**f**). Anterior regenerates (**a**) and posterior regenerates (**d**) fully healed the wound and build a blastema-like structure at the wound site. First, the original shape of the head (**b**) and the tail (**e**) was restored before the eyes (**c**) and the pigmentation (**c**,**f**) were regenerated. Arrows are pointing at the blastema-like tissue. Abbreviations: ey, eye. Scale bar is 100 µm in (**a**) and 50 µm in (**b**–**f**).

Complete wound healing was observed 1 dpa in anterior regenerates of *V. auriculatum* (Figure 7f,i) and 2 dpa in anterior regenerates of *P. girardi* (Figure 8b,e and Figure 9a). In both species, 94% of specimens reached this initial stage. During the following days, a blastema-like structure was formed and continued to grow in both species (Figure 10a). At the earliest after 6 dpa and at the latest after 8 dpa, the original head shape was rebuilt, which in *V. auriculatum* also included the small tentacles (Figure 7g,j, Figure 8c,f, Figure 9b and Figure 10b). This level of regeneration was reached by 75% of the initially amputated *V. auriculatum* and 62% of the *P. girardi* specimens. At 12 dpa, a pair of eyes was clearly visible in 60% of the amputees of *V. auriculatum* (Figure 7h,k and Figure 10c). In *P. girardi*, only 31% of the regenerates were able to regenerate eyes (Figure 8d,g and Figure 9c). Notably, of those specimens who formed eyes, only two thirds were able to regenerate two eyes. The remaining third was only able to regenerate a single eye.

**Figure 11:**
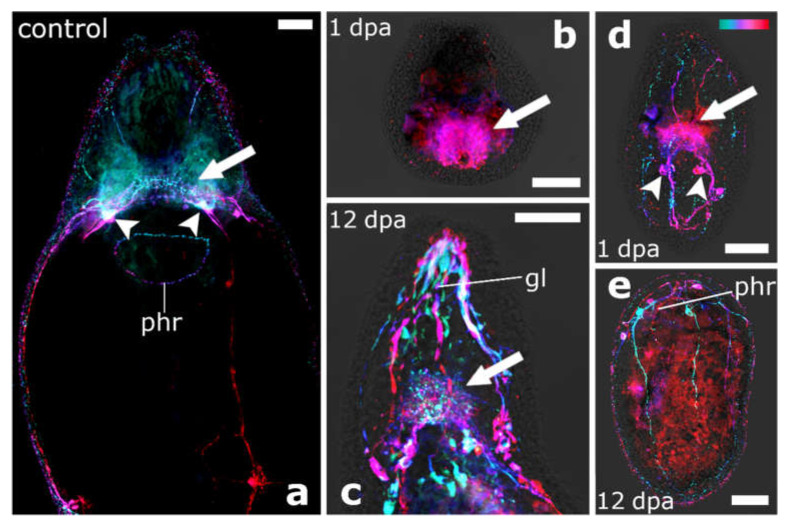
Staining of the serotonergic nervous system in anterior regenerates of *Vorticeros auriculatum*. (**a**) Intact animal (adapted from Figure 6b in Grosbusch et al., 2021). The presence of a brain in posterior (**b**) and anterior (**c**) regenerates of the same individual cut between brain and pharynx, fixed after 1 dpa and 12 dpa, respectively, indicates that the brain was regenerated in the anterior regenerate. Conversely, the presence of a brain in the posterior regenerate (**d**), but the absence of a brain in the anterior regenerate (**e**) of the same individual cut posterior of the pharynx, fixed after 1 dpa and 12 dpa, respectively, indicates that brain and pharynx were not regenerated in the anterior regenerate. Anterior is up in all images. All images are depth-coded and (**b**–**e**) are overlaying a brightfield image. Turquoise is more dorsal, red more ventral in (**b**,**c**), turquoise is more ventral, red more dorsal in (**d**,**e**). Arrowheads point to the brain roots while full arrows point to the neuropil. Abbreviations: gl, glands; phr, pharyngeal nerve ring. Scale bars are 50 µm.

Staining of the serotonergic nervous system in regenerates of *V. auriculatum* showed that only anterior regenerates still retaining the most posterior part of the brain (‘brain roots’) were able to regenerate the missing part of the brain (Figure 11a–c), while those regenerates without brain roots could not regenerate any part of the brain (Figure 11d–e). Anterior regenerates, which were able to regenerate a head, showed very prominent glands at the very front of the newly formed head, which were visible due to non-specific staining (Figure 11c).

##### Posterior and Anterior Regeneration in Other Plagiostomids

Four additional species of *Plagiostomum* were amputated between brain and pharynx to further explore regeneration capacity in this genus. The regeneration process in *Plagiostomum koreni* (*n* = 29) was similar to *P. girardi*. All posterior regenerates were unable to regenerate a tail during the two weeks of observation. In contrast, 48% of the anterior regenerates were able to rebuild the shape of the head after one week. Of those, one quarter was able to rebuild two eyes and one quarter was able to rebuild only a single eye. In *Plagiostomum chromogastrum* (*n* = 8), a single anterior regenerate, which was cut closer to the brain than the others, was able to regenerate the shape of the head and one eye at 17 dpa. Additionally, the anterior regenerates of *Plagiostomum maculatum* (*n* = 8) and *Plagiostomum morgani* (*n* = 9) were not able to regenerate the missing head during the two weeks of observation, but approximately half of the posterior regenerates of both species were able to regrow a tiny tail tip after one week.

#### 3.2.2. Amputation Posterior to the Pharynx

The amputation level posterior to the pharynx was analysed in specimens of *Vorticeros auriculatum* (*n* = 37) and *Plagiostomum girardi* (*n* = 41) (Figure 7l and Figure 8h).

##### Posterior Regeneration

Posterior regenerates of *V. auriculatum* showed a quick regeneration response after amputation. They were all (100%) able to heal the wound completely after around 18 h after the amputation and a blastema-like structure was built at the wound site (Figure 7m,q and Figure 10d) The blastema-like structure was slightly tapered at 2 dpa in 100% of the regenerates (Figure 7n,r and Figure 10e). Furthermore, all posterior regenerates had rebuilt the original body shape after no more than 4 dpa, but the pigmentation was still missing (Figure 7o,s). After one week, the tail tip had grown and the pigmentation was restored (Figure 7p,t and Figure 10f). During the following 5 days of observation, none of the regenerates died (Figure S2a) and the newly built tail tip continued to grow. In *P. girardi*, wound healing was completed after 2 dpa in 66% of posterior regenerates (Figure 8i,l and Figure 9d). A blastema-like structure was observed at 4 dpa in 61% of the posterior regenerates (Figure 8j,m and Figure 9e). This blastema-like structure continued to grow and taper until 10 dpa the original body shape was restored in 59% of the posterior regenerates (Figure 8k,n and Figure 9f). After total restoration of the body shape, amputees of both species were able to stick again to the ground with their new tail tip.

While in *V. auriculatum* there was no difference of the survival rate between amputation batches of the same cut level of posterior regenerates, in *P. girardi*, two batches, both amputated and observed in Innsbruck, showed a different survival rate (Figure S2c,d).

##### Anterior Regeneration

Anterior regenerates of *V. auriculatum* showed complete wound healing after 1 dpa and *P. girardi* after 2 dpa (data not shown). No other regeneration was observed in the following two weeks of observation.

Additionally, in the survival rate of anterior regenerates, there was a great difference between batches of *P. girardi*.

##### Posterior and Anterior Regeneration in Other Plagiostomids

The capacity to regenerate a tail when the posterior regenerate has an intact brain and pharynx was investigated in six other plagiostomid species (*Plagiostomum koreni* (*n* = 20), *Plagiostomum chromogastrum* (*n* = 7), *Plagiostomum* sp. (*n* = 10), *Plagiostomum maculatum* (*n* = 3), *Plagiostomum morgani* (*n* = 1) and *Acmostomum dioicum* (*n* = 30)). The anterior regenerates were unable to regrow the missing body part and the posterior regenerates were completely regenerated after approximately ten days. Only in a single species, *Plagiostomum* sp., a blastema-like structure that was bent towards the ventral side of the animal was observed while moving sluggishly through the Petri dish.

### 3.3. Regeneration Capacity in Protomonotresidae

#### 3.3.1. Amputation between Brain and Pharynx

**Figure 12:**
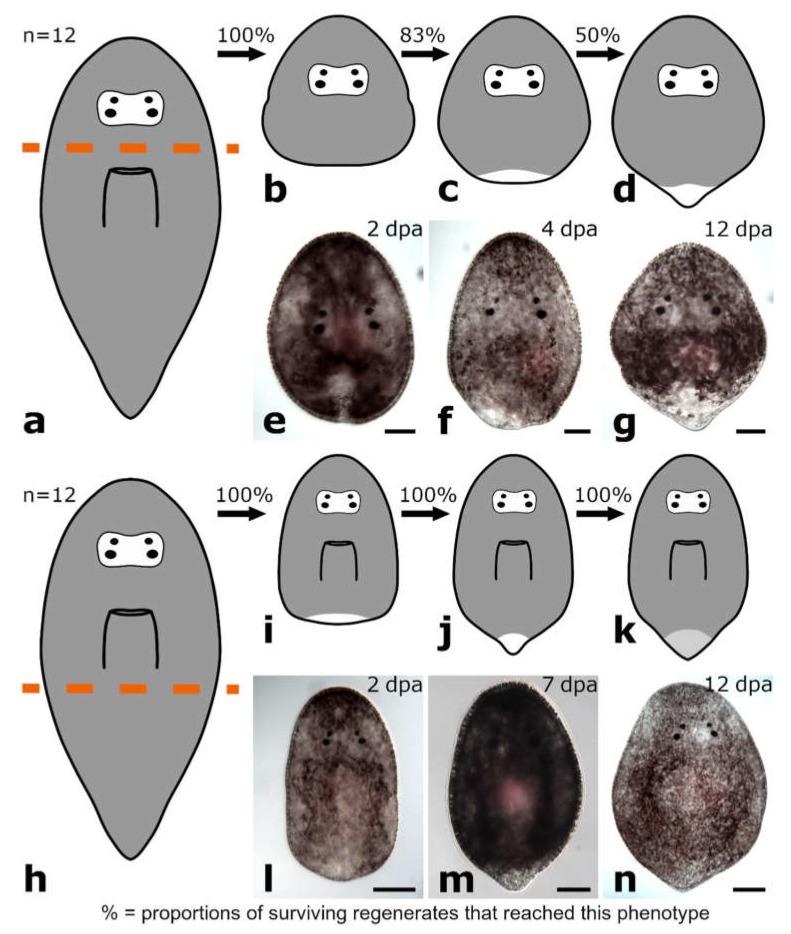
Regeneration in Protomonotresidae sp. Posterior regeneration after amputation between brain and pharynx (**a**–**g**). The wound was completely healed (**b**,**e**). A small, colourless blastema-like structure appeared (**c**,**f**) and developed into an unpigmented, pointy tail tip (**d**,**g**). Posterior regeneration after amputation posterior to the pharynx (**h**–**n**). The wound was fully healed and a small blastema-like structure appeared (**i**,**l**), which developed into a small pointy tail that lacked pigments (**j**,**m**). Pigments were restored in the regenerated tissue (**k**,**n**). Scale bars: 100 µm in (**l**,**n**); 50 µm in (**e**–**g**,**m**).

The amputation level between brain and pharynx was performed in specimens of Protomonotresidae sp. (*n* = 12) (Figure 12a).

##### Posterior Regeneration

**Figure 13:**
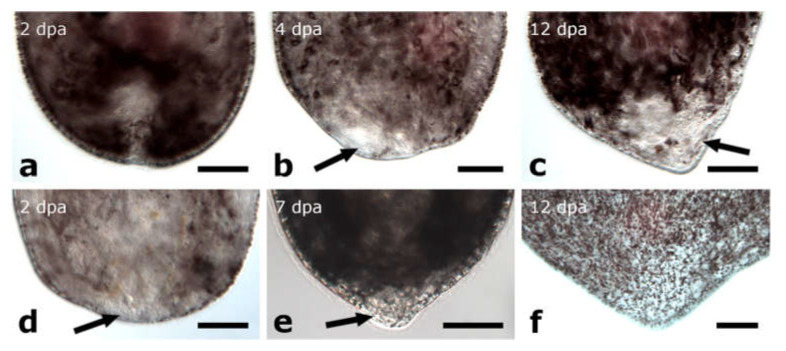
Blastema-like tissue in posterior regenerates of Protomonotresidae sp. After being amputated between brain and pharynx (**a**–**c**), the wound was fully healed (**a**) and an unpigmented, blastema-like structure was formed at the wound site (**b**). Finally, a new pointy tail tip was built, which was lacking the original pigmentation (**c**). Amputation posterior to the pharynx (**d**–**f**) After the wound was completely healed, an unpigmented, blastema-like structure was observed at the wound site (**d**). Few days later, the structure was tapered but it was still lacking the original pigmentation (**e**). One week later, also the pigmentation was restored (**f**). Arrows are pointing at the blastema-like tissue. Scale bar is 50 µm.

The wound was completely healed in 100% of posterior regenerates at 2 dpa (Figure 12b,e and Figure 13a). Four dpa, a small blastema-like structure at the amputation site became visible in 83% of posterior regenerates (Figure 12c,f and Figure 13b). Over the course of the observation time of two weeks, the blastema-like structure constantly grew and gradually tapered until 12 dpa 50% of the posterior regenerates looked like miniatures of intact animals with the only difference that the new tail tip lacked pigmentation (Figure 12d,g and Figure 13c).

##### Anterior Regeneration

In all anterior regenerates, wound healing was observed after 2 dpa, but no further changes were observed after that (data not shown). However, the regenerates were still alive at 13 dpa.

#### 3.3.2. Amputation Posterior to the Pharynx

The amputation level posterior to the pharynx was studied in specimens of Protomonotresidae sp. (*n* = 12) (Figure 12h).

##### Posterior Regeneration

In 100% of posterior regenerates, the wound was healed at 2 dpa and a blastema-like structure became visible (Figure 12i,l and Figure 13d). The blastema-like structure constantly grew and gradually tapered until 7 dpa, all regenerates looked like intact animals again, only the pigmentation was missing in the regenerative tissue (Figure 12j,m and Figure 13e). First pigmentation in the regenerated tail was observed in 100% of posterior regenerates after 9 dpa, which became stronger during the following days (Figure 12k,n and Figure 13f). However, it still remained weaker than in wild-type animals. Additionally, the newly built tail tip was not as long and tapered as originally.

##### Anterior Regeneration

Anterior regenerates showed a complete wound healing after 2 dpa, but no further regeneration was observed during the following 11 days (data not shown).

### 3.4. Molecular Phylogeny

**Figure 14:**
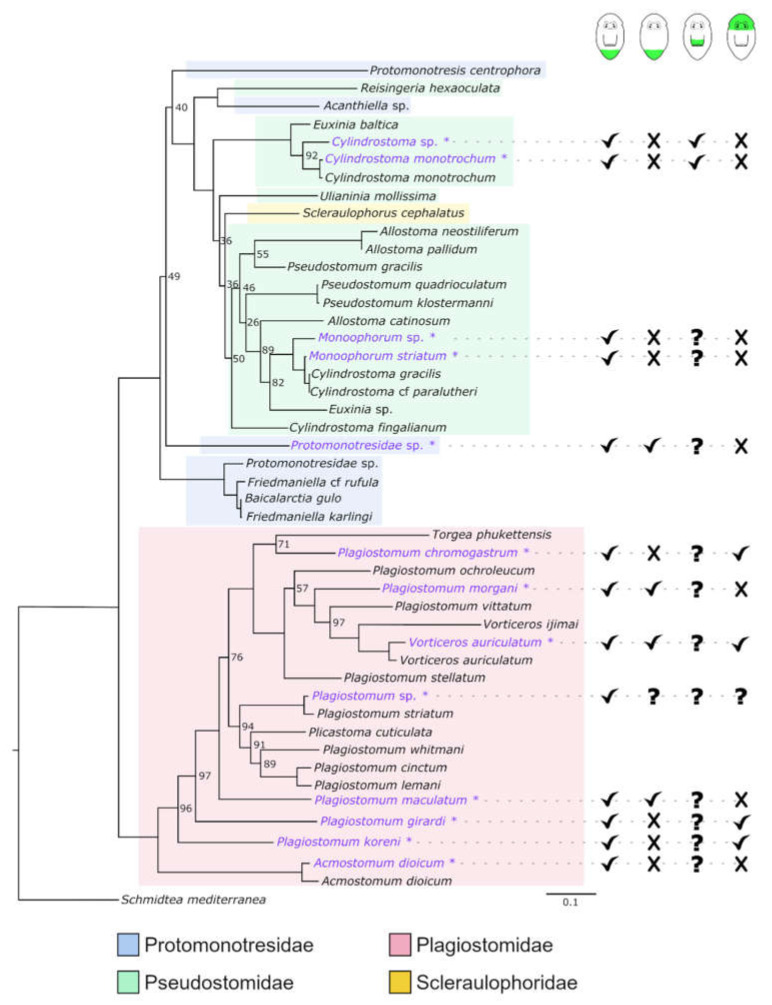
Maximum-likelihood reconstruction of Prolecithophora from partial 18S and 28S sequences, rooted with the triclad *Schmidtea mediterranea*. New sequences provided in this work are written in purple. Node numbers indicate bootstrap (bs) support values, bs support of 100 is omitted. Current classification in families is indicated by a coloured background; note that Protomonotresidae and Pseudostomidae do not appear to be monophyletic. Overall regeneration capacity as found in this study is indicated with check marks (=present), cross marks (=not present) or question marks (=not studied) for four categories (from left to right): (1) posterior regeneration takes place when the pharynx is present, (2) posterior regeneration takes place also in the absence of the pharynx, (3) parts of the pharynx can regenerate, and (4) parts of the head, including parts of the brain, can regenerate. Analysed species are marked with an asterisk.

Our phylogenetic reconstruction of Prolecithophora (Figure 14) places all 13 species used for regeneration experiments in the present work in the expected families, even if Protomonotresidae and Pseudostomidae are not recovered monophyletically, which correlates with low support values for these groups. In addition, many genera appear to be non-monophyletic: *Pseudostomum*, *Cylindrostoma*, *Monoophorum*, *Allostoma*, *Euxinia* (Pseudostomidae), *Plagiostomum* (Plagiostomidae) and *Friedmaniella* (Protomonotresidae).

## 4. Discussion

### 4.1. Comparative Regeneration in Prolecithophora

Regeneration in Prolecithophora is only superficially and seemingly contradictorily covered in the literature. Keller [22] mentions *Plagiostomum lemani* as an example of a turbellarian flatworm with low regeneration capacity, but does not give further details. Contrastingly, Monti [23] writes that its congener *Plagiostomum girardi* can regenerate all missing body parts and organs after being bisected either transversely or longitudinally, but does not provide figures or any details. Our own results show that both authors may be correct, as it depends on both, species and amputation level, whether plagiostomid species are able to regenerate specific body parts (Figure 5 and Figure 6). The time needed until full regeneration of seven to ten days reported for *P. girardi* [23] is in line with our own observations.

As shown in the present study, the regeneration capacity of different species of prolecithophorans covers a broad range: from being barely able to regenerate a rather minuscule posterior tip (*Monoophorum striatum*), over the ability to regenerate a part of the pharynx (*Cylindrostoma monotrochum*), to the ability to regenerate the greater part of the brain and the eyes (e.g., *Vorticeros auriculatum* and *Plagiostomum girardi*). Thereby, regeneration capacity reflects family affiliations.

**Figure 15:**
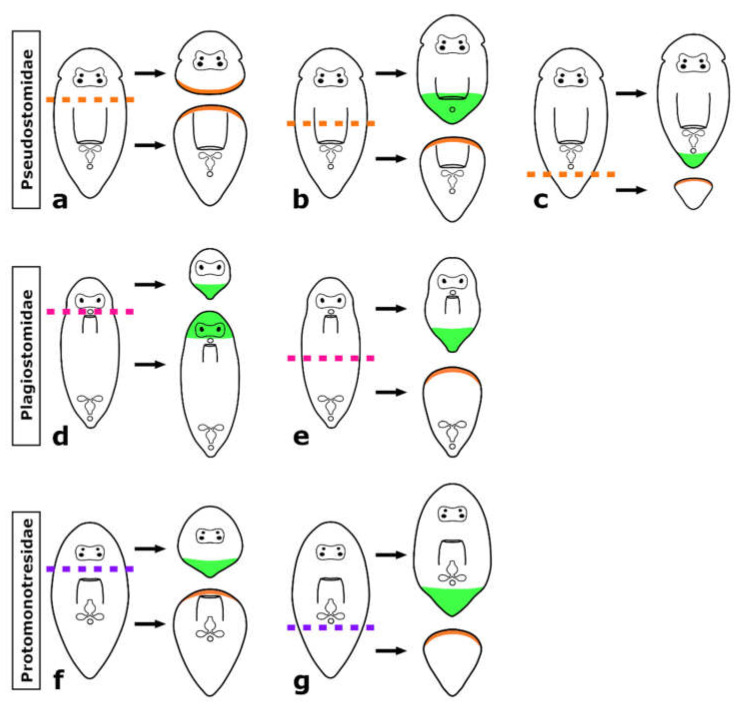
Regeneration capacity in Prolecithophora. Summary of the best known regeneration capacity in Pseudostomidae (**a**–**c**), Plagiostomidae (**d**,**e**) and Protomonotresidae (**f**,**g**) after amputation between brain and pharynx (**a**,**d**,**f**), through the pharynx (**b**) and posterior to the pharynx (**c**,**e**,**g**). Green highlights in the schematics show regenerated tissues while orange highlights indicate only wound healing.

First, the three studied pseudostomid species cannot regenerate any part of the anterior body, i.e., the head containing brain and eyes (Figure 14 and Figure 15a). However, they are able to regenerate a posterior part of the body, but only if at least a part of the pharynx is still present (Figure 14 and Figure 15b,c). Next, the eight studied plagiostomid species can rebuild a posterior body part (Figure 14 and Figure 15d,e) and while some species need the pharynx to be present, others do not. They are the only group of prolecithophorans able to regenerate a head including the brain and eyes, but two conditions must be met: a) the pharynx needs to be intact and b) the amputation needs to be made directly behind the main part of the brain, so that the brain roots remain in the regenerating piece (Figure 14 and Figure 15d). Finally, the single studied protomonotresid species is able to regenerate a tail tip regardless of the presence of the pharynx, but it cannot regenerate any anterior body parts (Figure 14 and Figure 15f,g). Unfortunately, it is not clear whether it is able to regenerate a new pharynx or not.

Interestingly, not only the regeneration capacity varies greatly between prolecithophorans, but also the speed at which they regenerate. While *Cylindrostoma* and *Monoophorum* need approximately two weeks to regenerate the posterior body part, protomonotresids and plagiostomids achieve this in approximately half the time. In summary, Pseudostomidae regenerate slowly, poorly and only posteriorly, Protomonotresidae regenerate faster, but still poorly and only posteriorly, while Plagiostomidae regenerate faster, better and often anteriorly (see Figure 14 and Figure 15 for an overview).

In *C. monotrochum* and *P. girardi*, survival rates of the same amputation level were not consistent between batches while in the remaining three species no difference was noticed. However, the causes for these differences do not seem to be the same. *C. monotrochum* seems to be sensitive to long transportation times as the survival curve of batches cut and observed in the field was higher than that of the batches studied in the remote laboratory in Innsbruck. As for *P. girardi,* the environmental conditions seem to be crucial as only one batch sampled in June 2018 showed a different survival curve.

### 4.2. Molecular Phylogeny

The phylogenetic reconstruction shown in Figure 14 closely reflects the overall tree topology of [25], with the omission of Genostomatidae, which are currently not classified as prolecithophorans, but as a possible sister group to Prolecithophora [25]. Our new sequences provided for *Cylindrostoma monotrochum*, *Vorticeros auriculatum* and *Acmostomum dioicum* are recovered as sister groups of published sequences of the same species, which indicates that our morphological species determination was correct. Four species used in this study were not sufficiently morphologically determined: *Cylindrostoma* sp., *Monoophorum* sp., *Plagiostomum* sp. and Protomonotresidae sp., and await further determination or description. We provided the first genetic data for ten species, which were also recovered in expected parts of the tree, with the exception of *Monoophorum* sp., which is not sister group of *Monoophorum striatum*, but is closely related. The non-monophyly of two families and seven genera calls for a revision of Prolecithophora as a whole.

There is no clear pattern for regeneration capacity in different groups of Prolecithophora, with the exception of partial head regeneration, which is restricted to Plagiostomidae.

### 4.3. Regeneration Capacity Is Linked to the Shape and the Size of the Regenerate

The difference in regeneration capacity may be related to the difference in the morphology between prolecithophoran families and species. Plagiostomids have a more flattened body shape than pseudostomids and protomonotresids. Although protomonotresids have a rounder body shape than plagiostomids, they are not as broad as pseudostomids [28,38,48]. This leads to a smaller wound site in plagiostomids and protomonotresids and thus to a lower risk of losing cellular mass immediately after amputation than in pseudostomids. The size of the wound site could be related to the regeneration time, a smaller wound site leads to a faster regeneration process. Besides Pseudostomidae, Plagiostomidae and Protomonotresidae, the taxon Prolecithophora encompasses two other families, Multipeniatidae and Scleraulophoridae, which were not analysed in this study. While Multipeniatidae are described as morphologically very similar to the Plagiostomidae, Scleraulophoridae are more similar to Pseudostomidae [30,34,49]. Thus, based on the morphology, we hypothesise that the regeneration capacity of Multipeniatidae is similar to that of Plagiostomidae, while that of Scleraulophoridae is more similar to that of Pseudostomidae.

In the triclad *Girardia dorotocephala* and the macrostomorphan *Macrostomum lignano*, the minimal cell number was determined where regeneration is still possible [15,50]. The small size of the regenerates may also play a role in plagiostomids, where species with more remaining cell material were found to be able to regenerate posteriorly, while species with less material were not (Figure S3). A similar case can be made for pseudostomids, which are only able to regenerate part of the pharynx posteriorly in species, where the resulting posterior regenerate is bigger (*Cylindrostoma* spp.) than in species, which cannot (*Monoophorum* spp.). Additionally, maintaining the correct body proportions is an important part of the regeneration process in triclad flatworms [51]. The maintenance of proper body proportions of the regenerate was also observed in *C. monotrochum*. When amputated in the middle of the body the restored pharynx had a diameter of ca. 99 +/− 7.5 µm, while the original size is ca 150 +/− 6.4 μm [52].

### 4.4. The Brain Root Is Required for Head Regeneration

In the present study, three out of six plagiostomid species were able to regenerate a head with an intact brain and eyes if amputated between brain and pharynx. Stainings of the serotonergic nervous system of regenerates of *V. auriculatum* revealed that only anterior regenerates with brain roots, which contain an accumulation of perikarya of cerebral neurons, were able to regenerate a brain with eyes. Therefore, we state the hypothesis that the brain roots are necessary to regenerate the main part of the brain in plagiostomids. A recent study on triclads showed that three populations of guidepost-like cells play a decisive role during axon regeneration. While two populations of guidepost-like cells are muscle cells, the third are neurons positioned close to a decision point for axons [53]. Consequently, the neurons that form the brain roots of plagiostomids could possibly serve as guidepost-like cells in the regeneration of the brain. Regeneration studies on two polyclad species (*Theama mediterranea* and *Prosthiostomum siphunculus*), a proseriate species (*Otomesostoma auditivum*) as well as on *Macrostomum lignano* also showed that part of the brain must remain intact for anterior regeneration to be possible [15,18,19,54]. This suggests that certain cells of the brain also potentially act as guidepost-like cells in other flatworm species.

Stainings of the serotonergic nervous system in intact animals of *P. girardi* and *P. koreni* showed that the general pattern of the brain was the same as in *V. auriculatum*. On the left and right posterior tip of the brain were distinct brain roots with accumulation of perikarya of cerebral neurons [55] (Figure S4). Additionally, histological sections of the six studied plagiostomid species showed that the distance between brain and pharynx varied. In *V. auriculatum*, *P. girardi* and *P. koreni*, the pharynx was closer to the brain than in the other three species [37] (Figure S3). Therefore, the probability of brain roots remaining in the anterior regenerate after an amputation between brain and pharynx is higher in *V. auriculatum*, *P. girardi* and *P. koreni* than in other plagiostomid species. Consequently, more anterior regenerates of these three species were able to regenerate a brain. Only a single individual of *P. chromogastrum* which was cut closer to the brain, was able to regenerate a brain, while all other regenerates that were cut at an increased distance to the brain, were not. This finding speaks in favour of our hypothesis that brain roots are necessary to regenerate the brain.

None of the analysed pseudostomid species nor the studied Protomonotresidea sp. was able to regenerate a brain. In pseudostomids, this may be due to a distinct brain capsule surrounding the brain, which is absent in plagiostomids and protomonotresids [28,34,48,55]. Additionally, stainings of the serotonergic nervous system showed that prominent brain roots are found at the posterior part of the plagiostomid brain, whereas these are absent in pseudostomids [55]. Thus, we assume that the brain capsule also encloses the brain roots and therefore no part of the brain remains in the anterior regenerates in pseudostomids. In the studied Protomonotresidae sp., there is a greater distance between brain and pharynx, which possibly results in the brain roots remaining entirely in the posterior regenerate. Since protomonotresids are described without a brain capsule and descriptions of the brain structure are similar to those of plagiostomids [56], they are most likely able to regenerate the brain, if amputated right behind the brain.

### 4.5. Why Can the Pharynx Not Regenerate de Novo?

It may seem curious that some plagiostomids can regenerate a head, but not a pharynx. However, this conundrum can be explained by the specific anatomy of these animals. An anteriorly directed pharynx close to the brain leaves only a relatively short posterior regenerate (Figure 14 and Figure 15), which might be too small that organ regeneration can occur within. In anteriorly regenerating pieces of any tested prolecithophoran, a pharynx was never regenerated as well, possibly due to missing clues from the absent brain, as has been hypothesised in *Macrostomum lignano* [15]. Due to a posteriorly directed pharynx at approximately mid-body level, a partial pharynx regeneration could be observed in pseudostomids (Figure 14 and Figure 15), while in protomonotresids, it remained unclear if any part of the pharynx was regenerated. In summary, de novo regeneration of the pharynx in Prolecithophora seems to be limited by the size of the regenerate and/or by the lack of the brain.

### 4.6. Comparative Regeneration in Platyhelminthes

The regeneration capacity of most studied species belonging to the Prorhynchida and the Rhabdocoela is relatively poor. While some species cannot regenerate at all, some are at least able to restore a small posterior tip [20], very similar to eight of the thirteen here analysed species, namely the pseudostomid species *Monoophorum striatum* and *Monoophorum* sp., the undetermined protomonotresid species, and the plagiostomid species *Acmostomum dioicum*, *Plagiostomum* sp., *Plagiostomum koreni*, *Plagiostomum maculatum*, and *Plagiostomum morgani*.

The macrostomorphan *Macrostomum lignano* cannot regenerate the brain and only approximately half of the pharynx [15]. Regeneration capacity of *Cylindrostoma monotrochum* and *Cylindrostoma* sp. observed in this study is most similar to the one described for *Macrostomum*. Additionally, similar to *M. lignano* [57], the early blastema in *C. monotrochum* and *Plagiostomum* sp. are bent to the ventral side. Since it is not easy to detect a ventrally bent blastema in live animals, it may be a more common feature than currently appreciated.

Polyclads cannot regenerate a complete brain, but several species have been found to be able to regenerate the anterior half of the brain, but not the posterior half [18,19]. For the freshwater proseriate *Otomesostoma auditivum*, it was reported that amputations at or immediately posterior to the brain lead to partial restoration of the brain, eyes and sensory pits [54]. These findings are reminiscent of the plagiostomid species *Vorticeros auriculatum*, *Plagiostomum girardi*, *Plagiostomum koreni* and *Plagiostomum chromogastrum*, in which brain regeneration is only possible in the presence of (posterior) brain roots (see chapter 4.3).

The variable regeneration capacity of different prolecithophorans is also found in other flatworm taxa. In polyclads, the regeneration capacity ranges from being able to regenerate only small lateral tissues, to being able to regenerate the pharynx and parts of the brain [18,19]. A similar picture is presented in macrostomorphans, where some species can regenerate all body parts (e.g., *Microstomum*), while others cannot regenerate the complete head or the complete pharynx (e.g., *Macrostomum lignano*). Interestingly, the ability to regenerate a head has likely convergently evolved several times within Macrostomorpha [58].

### 4.7. Is the Capacity to Regenerate All Organs an Ancestral Feature in Adiaphanida?

Species in most higher-level flatworm taxa cannot regenerate a head (Figure 1). Exceptions are known from only three out of approximately a dozen higher-level flatworm taxa: Catenulida, Macrostomorpha and Tricladida [20]. As Tricladida are only distantly related to Catenulida and Macrostomorpha, it seems most parsimonious to consider head regeneration capacity in Tricladida as a convergent feature. The open question was whether head regeneration capacity is only convergent in Tricladida or in all Adiaphanida. The patchy and always deficient regeneration capacity found throughout the studied prolecithophorans [22,23] (Figure 14 and Figure 15) strongly suggests that the adiaphanidan ancestor lacked a complete head regeneration capacity. So far, nothing is known about the regeneration capacity of the remaining adiaphanidan taxon, Fecampiida.

## 5. Conclusions

To expand the sparse knowledge about the regeneration capacity of Prolecithophora, the possible sister group of Tricladida, we analysed 13 different species among three families (Pseudostomidae, Plagiostomidae, Protomonotresidae). We showed that all studied species can regenerate a tail if amputated posterior to the pharynx. However, some species belonging to the Plagiostomidae were able to regenerate a head, including brain and eyes, but only if the brain roots were still present in the anterior regenerate. Furthermore, partial pharynx regeneration could be observed only in two pseudostomid species belonging to the same genus. The difference in regeneration capacity is possibly related to the different morphology of the species, e.g., the distance between brain and pharynx, which greatly affects the remaining body size of the regenerates at the studied cutting levels.

Although Tricladida is closely related to Prolecithophora, their regeneration capacity is not the same. The studied prolecithophorans show higher similarity to the regeneration capacity found in macrostomids, polyclads, and proseriates. This suggests that triclads have independently evolved the capacity to completely regenerate the head.

## Data Availability

The sequences were deployed at NCBI and accession numbers can be found in Table S1 in the supplementary.

## Supplementary Materials

The following supporting information can be downloaded at: https://www.mdpi.com/article/10.3390/biology11111588/s1, Figure S1: Survival curves of regenerates of *Monoophorum striatum* and *Cylindrostoma monotrochum*; Figure S2: Survival curves of regenerates of *Vorticeros auriculatum* and *Plagiostomum girardi***;** Figure S3: Histological sections of intact animals of all analysed plagiostomid species; Figure S4: Serotonergic nervous system of intact animals of *P. koreni* and *P. Girardi*, Table S1: Sequences used for phylogenetic reconstruction shown in Figure 14.
