# Supplementary material for: Do Not Lose Your Head over the Unequal Regeneration Capacity in Prolecithophoran Flatworms
Source: Biology (Basel). 2022 Oct 28;11(11):1588. doi: 10.3390/biology11111588 (PMC9687166; doi:10.3390/biology11111588)
Supplement: Supplementary file 1 [file biology-11-01588-s001.zip › biology-1931342-supplementary.pdf]

SUPPLEMENTARY FILES

Don’t lose your head over the unequal regeneration capacity in prolecithophoran flatworms

Alexandra L. Grosbsuch[1], Philip Bertemes[1], Bob Kauffmann[1], Clemens Gotsis[1] and Bernhard Egger[1,*]

Submitted to Biology

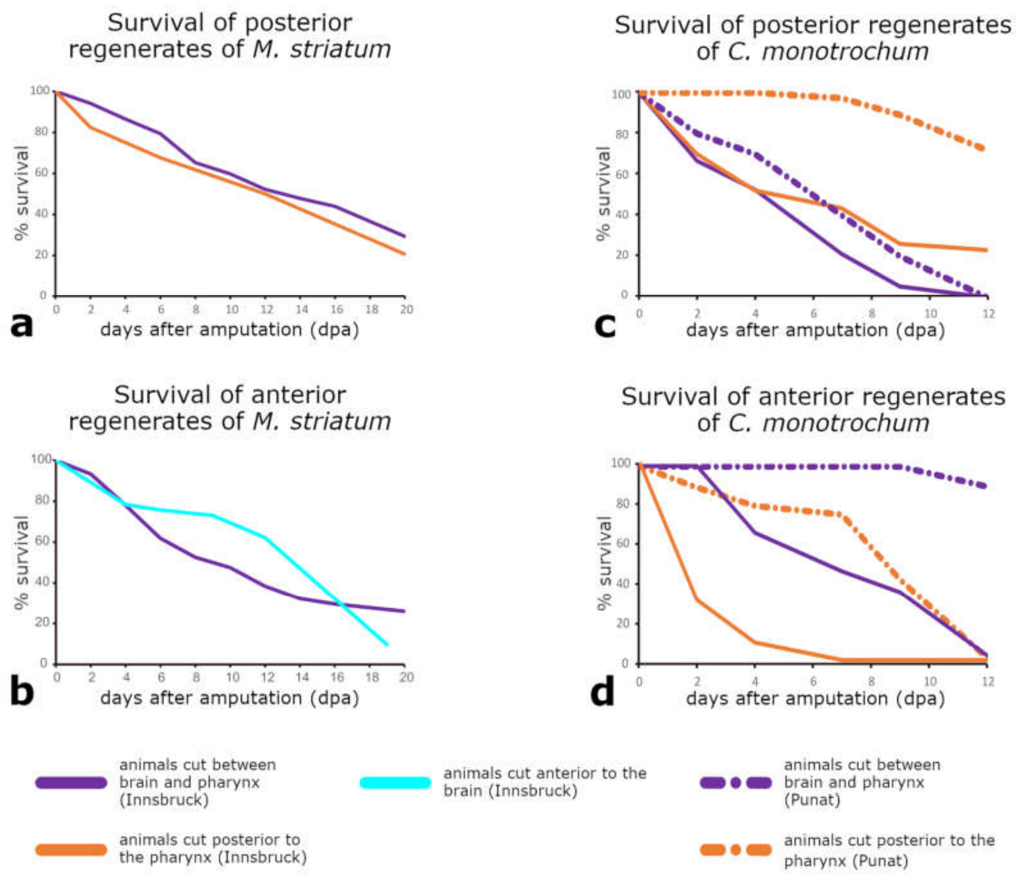

Figure S1: Survival curves of regenerates of *Monoophorum striatum* and *Cylindrostoma monotrochum*. Survival rates of posterior (**a**) and anterior regenerates (**b**) of *M. striatum* over the course of 20 days after amputation at their respective amputation level. Survival rates of posterior (**c**) and anterior regenerates (**d**) over the course of 12 days after amputation at their respective amputation level. Note that dashed lines were regeneration experiments carried out in the field (animals fresh from the sea and daily fresh sea water vs. animals that had to be transported to the facilities in Austria and were kept in artificial sea water).

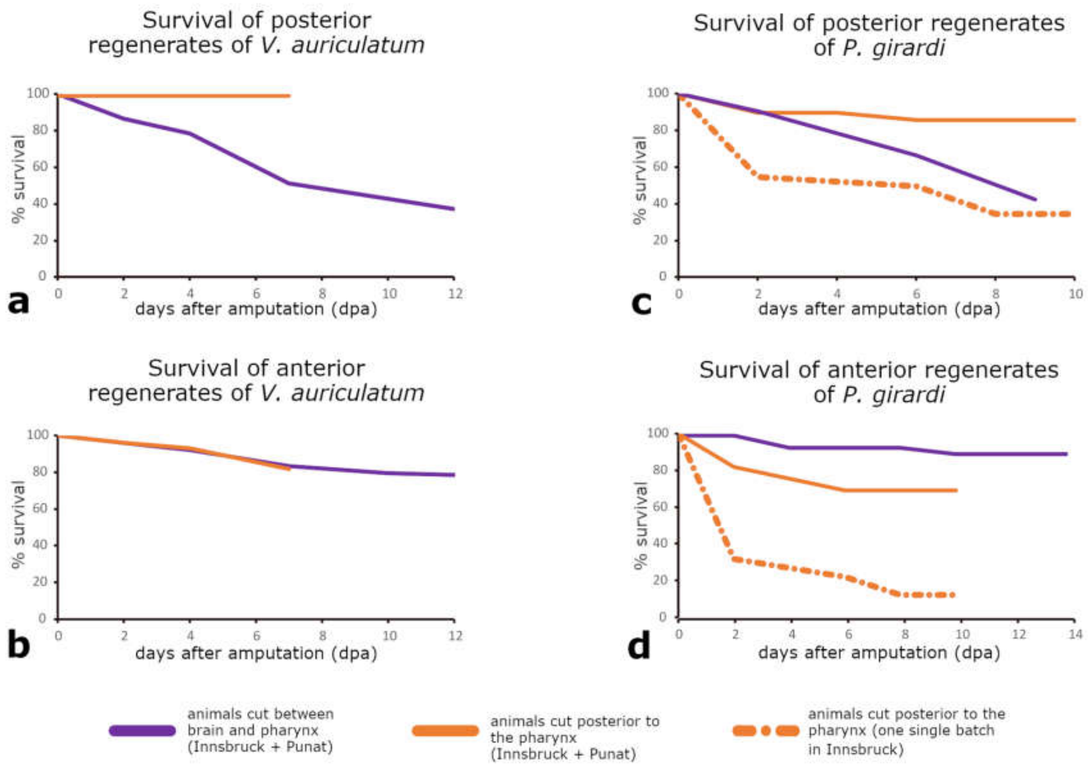

Figure S2: Survival curves of regenerates of *Vorticeros auriculatum* and *Plagiostomum girardi*. Survival rates of posterior (**a**) and anterior regenerates (**b**) of *V. auriculatum* over the course of 12 days after amputation at their respective amputation level. Survival rates of posterior (**c**) and anterior regenerates (**d**) of *P. girardi* over the course of 10 and 14 days after amputation at their respective amputation level. Dashed lines represent a regeneration experiment carried out in the lab in Innsbruck, where the only deviating survival rates were found.

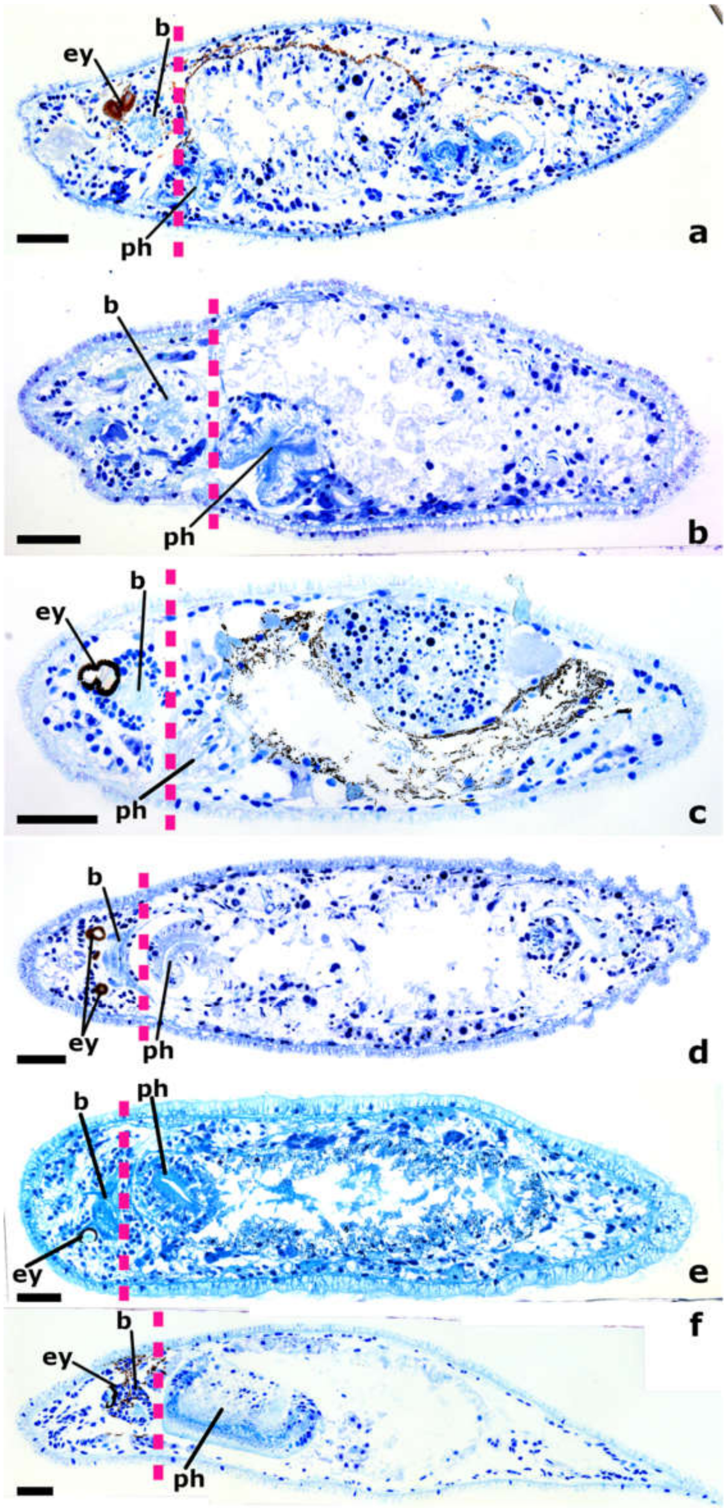

Figure S3: Histological sections of intact animals of all analysed plagiostomid species. Bright field image of histological sections stained with toluidine blue of *V. auriculatum* (**a**), *P. morgani* (**b**), *P. chromogastrum* (**c**), *P. koreni* (**d**), *P. girardi* (**e**) and *P. maculatum* (**f**). **a** - **c** and **f** are lateral views, **d** - **e** are dorsal views. Anterior is left for all animals. The pink, dashed lines indicate the amputation level. Abbreviations: b, brain; ey, eyes; ph, pharynx. Scale bar is 50 µm.

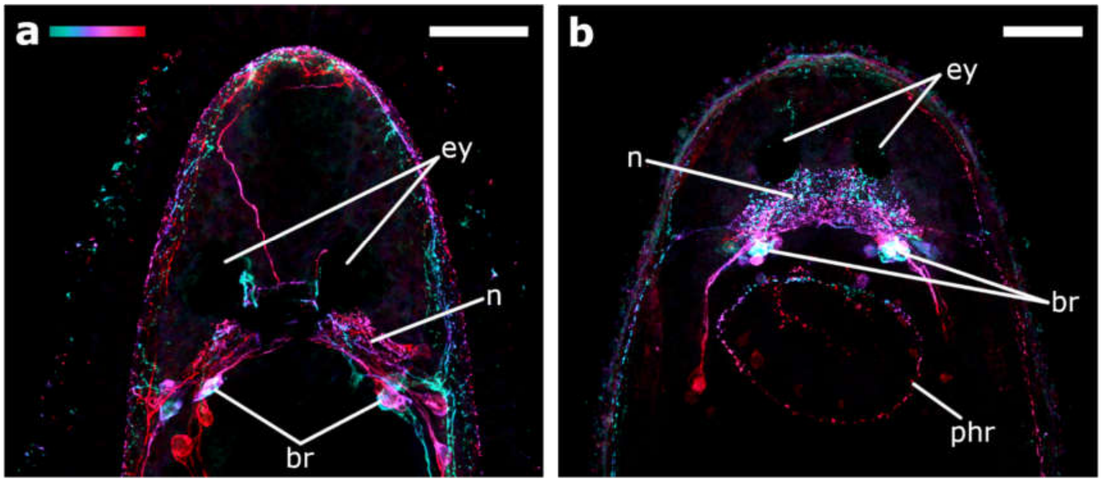

Figure S4: Serotonergic nervous system of intact animals of *P. koreni* and *P. girardi*. Depth colour coded image of a dorsal view of the head region of *P. koreni* (**a**) and *P. girardi* (**b**). Turquoise is more dorsal, red more ventral. Anterior is up for all animals. Abbreviations: br, brain roots; ey, eyes; n, neuropile; phr, pharyngeal nerve ring. Scale bar is 50 µm.

Table S1. Sequences used for phylogenetic reconstruction shown in Fig. 14. Bold species names indicate new sequences provided for this publication.

Family	Species name	18S accession number	28S accession number
Dugesiidae	*Schmidtea mediterranea*	U31084.1	DQ665992.1
Plagiostomidae	***Acmostomum dioicum***	OP627012	OP626914
	Acmostomum dioicum	AF503516.1	
	Plagiostomum chromogastrum	OP627003	
	Plagiostomum cinctum	AF065418.1	AJ313224.1
	Plagiostomum girardi	OP627009	OP626912
	Plagiostomum koreni	OP627005	OP626904
	Plagiostomum lemani	AF503518.1	
	Plagiostomum maculatum	OP627006	OP626909
	Plagiostomum morgani	OP627007	OP626906
	Plagiostomum ochroleucum	AF065419.1	AJ313225.1
	***Plagiostomum* sp.**	OP627004	OP626905
	Plagiostomum stellatum	KC869819.1	KC869872.1
	Plagiostomum striatum	AF065420.1	AJ313226.1
	Plagiostomum vittatum	AF065421.1	AJ313227.1
	Plagiostomum whitmani	KC869818.1	KC869871.1
	Plicastoma cuticulata	AF065422.1	AY157158.1
	Torgea phukettensis	AF503517.1	
	Vorticeros auriculatum	OP627011	OP626913
	Vorticeros auriculatum	AJ312267.1	AJ313229.1
	Vorticeros ijimai	D85094.1	
Protomonotresidae	*Acanthiella* sp.	KC869786.1	KC869839.1
	Baicalarctica gula	AJ287483.1	
	Friedmaniella cf *rufula*	AJ287512.1	
	Friedmaniella karlinigi	AJ287513.1	
	Protomonotresis centrophora	AF167419.1	AJ313218.1
	***Protomonotresidae* sp.**	OP627000	OP626908
	Protomonotresidae sp.	KC869820.1	KC869873.1
Pseudostomidae	*Allostoma catinosum*	AJ312267.1	MK791299.1
	Allostoma neostiliferum	AF167420.1	
	Allostoma pallidum	AJ312265.1	AJ313214.1
	Cylindrostoma fingalianum	AF065415.1	AJ313215.1
	Cylindrostoma gracilis	AF065416.1	AJ313216.1
	Cylindrostoma monotrochum	OP627002	OP626907
	Cylindrostoma monotrochum		AJ313217.1
	Cylindrostoma cf *paralutheri*	AJ312266.1	AJ313223.1
	***Cylindrostoma* sp.**	OP627001	OP626910
	Euxinia baltica	AF167418.1	
	Euxinia sp.		MK791291.1
	Monoophorum striatum	OP627008	OP626911
	***Monoophorum* sp.**	OP627010	OP626915
	Pseudostomum gracilis	AF065423.1	

	Pseudostomum klostermanni	AF065424.1	AJ313219.1
	Pseudostomum quadrioculatum	AF065425.1	
	Reisingeria hexaoculata	AF065426.1	AJ313220.1
	Ulianinia mollissima	AF065427.1	AJ313222.1
Scleraulophoridae	*Scleraulophorus cephalatus*	AF167423.1	AJ313221.1
